# Investigation of the inhibition effect of 1,2,3,4,6-pentagalloyl-β-D-glucose on gastric cancer cells based on a network pharmacology approach and experimental validation

**DOI:** 10.3389/fonc.2022.934958

**Published:** 2022-08-03

**Authors:** Jing-hui Bi, Yu-han Jiang, Shi-jie Ye, Min-rui Wu, Yang Yi, Hong-xun Wang, Li-mei Wang

**Affiliations:** ^1^ College of Life Science and Technology, Wuhan Polytechnic University, Wuhan, China; ^2^ College of Food Science and Engineering, Wuhan Polytechnic University, Wuhan, China

**Keywords:** β-PGG, gastric cancer, network pharmacology, mechanism, p53 signaling pathway

## Abstract

**Background:**

Gastric cancer (GC) is ranked as the third leading cause of cancer-related mortality worldwide. 1,2,3,4,6-Pentagalloyl-β-D-glucose (β-PGG) has various pharmacological activities and has been shown to suppress cancer development. However, the mechanism by which β-PGG inhibits gastric cancer has not been elucidated.

**Objective:**

This study explored the potential targets and mechanism of β-PGG in GC using the network pharmacology approach combined with *in-vitro* experiments.

**Methods:**

The PharmMapper software was used to predict the potential targets of β-PGG, and GC-related genes were identified on the GeneCards database. PPI analysis of common genes was performed using the STRING database. The potential regulatory mechanism of β-PGG in GC was explored through Gene Ontology (GO) and Kyoto Encyclopedia of Genes and Genomes (KEGG) pathway analyses. The binding ability of key genes and target proteins was verified by molecular docking. The effects of β-PGG on genes and proteins were evaluated using the CCK-8 assay, cell cycle analysis, apoptosis assay, real-time fluorescence quantification polymerase chain reaction (qRT-PCR), and Western blotting.

**Results:**

Eight hub genes involved in cell cycle progression and apoptosis were identified. Cancer-related signaling pathways were identified using the Cytoscape tool. Some of those genes were significantly enriched in the p53 signaling pathway. The CCK-8 assay showed that β-PGG inhibited the proliferation of GC cells. Cell cycle and apoptosis experiments revealed that β-PGG induced cell cycle arrest and apoptosis of gastric cancer cells. qRT-PCR and Western blot analysis showed that β-PGG inhibited β-PGG cells by modulating the p53 signaling pathway.

**Conclusion:**

In the present study, the targets and mechanism of β-PGG in gastric cancer were explored. The results indicate that β-PGG can be used to develop treatments for GC.

## Introduction

Cancer lowers the life expectancy of patients. The mortality rate of gastric cancer (GC) is about 7.7%, and it ranks fifth and third (1) in terms of cancer incidence and mortality rate globally, respectively. Most gastric cancers are diagnosed at an advanced stage and have a high risk of metastasis and recurrence after radical resection (2). Currently, the main treatment modalities for gastric cancer are surgical treatment (3, 4), adjuvant therapy, and targeted therapy (5). However, the clinical outcomes of these modalities are not satisfactory. Therefore, it is imperative to develop safe and effective adjuvant antitumor drugs.

1,2,3,4,6-Pentagalloyl-β-D-glucose (β-PGG) is a polyphenolic tannic acid monomer compound present in several plants such as water chestnut shells (6), *Paeonia lactiflora* Pall. (7), and *Elaeocarpus sylvestris* (8). PGG has anti-inflammatory, antidiabetic, and hepatoprotective effects (9); inhibits adipogenesis (10, 11), hemolysis, and methemoglobin formation (12); suppresses platelet activation (13); has antiviral and immunomodulatory activities (8, 14, 15); and decreases monophenolase activity (16). Previous studies reported that PGG can prevent the development of various cancers including colon cancer (17), breast cancer (18), pancreatic cancer (19), prostate cancer (20), glioma (21), liver cancer (22), chronic myelogenous leukemia (23), and kidney cancer (24). However, its mechanism and targets have not been elucidated.

Network pharmacology is a drug designing method comprising system biology, network analysis, connectivity, redundancy, and pleiotropy (25). This approach is based on the concept of “multitarget and multicomponent therapy,” which has higher therapeutic efficacy compared with “single-target therapy.” Investigating network relationships among drug–gene–disease interactions using network pharmacology can further reflect the multitarget and multichannel nature of drug therapy. This strategy plays a key role in the development of new drugs and the exploration of biological mechanisms. Zhou explored the anti-inflammatory effect of Xuebijing in the treatment of sepsis using network pharmacology (26). Moreover, Liu et al. (27) evaluated the antitumor effect of gossypol acetic acid on gastric cancer using the network pharmacology approach. Furthermore, Zheng et al. (28) explored the mechanism of traditional Chinese medicine in the treatment of endometriosis using network pharmacological methods. Currently, network pharmacology is in its infancy (29), and the identified targets should be verified further. The mechanism by which β-PGG inhibits gastric cancer is not clear. In addition, no study has used network pharmacology to study the mechanism of β-PGG in GC. Therefore, we explored the potential targets of β-PGG in GC. A “GC–target–β-PGG” network was constructed using the network pharmacology approach, and the mechanism of β-PGG against GC was explored. The findings showed that β-PGG inhibited cancer cells by inducing apoptosis and inhibiting the cell cycle. These results were verified through *in-vitro* experiments including cell assays, qRT-PCR, and Western blotting. The findings of the present study provide a basis for follow-up research and development of novel drugs for the treatment of GC.

## Materials and methods

### Materials and reagents

β-PGG (purity ≥ 98%) was purchased from Shanghai Tongtian Biology Co., Ltd., China (Article No. e-1144). Dulbecco’s modified Eagle medium (DMEM) was purchased from HyClone, USA; penicillin–streptomycin mixture (100×) double antibody and fetal bovine serum were from Sijiqing Bioengineering Materials Company, Hangzhou, China; trypsin and CCK-8 kits were from White Shark Biotechnology Co., Ltd., Hefei, China; and DNA content detection kit, mitochondrial membrane potential detection kit (JC-1), and calcium ion detection kit were from Solarbio Technology Co., Ltd., Beijing, China. Annexin V-FITC apoptosis detection kit was from Beibo Biotechnology Company, Shanghai, China; TRIzol reagent, reverse transcription kit, and fluorescence quantitative kit were bought from Baoriyi Biotechnology (Beijing) Co., Ltd., Beijing, China; and T25 flask, 96-well plate, cryopreservation tube, and six-well plate were purchased from Corning Company Co., Ltd, USA. The following Western blot-related antibodies were purchased from Proteintech Group, Inc. (Wuhan, China): BAX polyclonal antibody (21KD; Cat No. 50599-2-Ig), BCL-2 polyclonal antibody (26KD; Cat. No. 26593-1-AP), and P21 polyclonal antibody (21KD; Cat. No. 10355-1-AP). Anti-β-actin antibody (42KD; Cat. No. BM0627), HRP-conjugated AffiniPure goat anti-mouse IgG (H+L; Cat. No. BA1050), and HRP-conjugated AffiniPure goat anti-rabbit IgG (H+Ll; Cat No. BA1054) were purchased from Wuhan Boster Biological Technology Co., Ltd. Caspase 9 antibody (46KD; Cat. No. AF6348), cleaved-caspase 3 (Asp175), p17 antibody (17KD; Cat. No. AF7022), caspase 3 antibody (35KD; Cat. No. AF6311), and BBC3 antibody (20KD; Cat. No. AF5173) were purchased from Affinity Biosciences Co., Ltd. p53 (DO-7) and mouse mAb (53KD; Cat. No. 48818S) were purchased from Cell Signaling Technology, Inc. (CST, USA).

### Instruments and equipment

The following were used in the study: Sim CO_2_ incubator [Ximeng (SIM) Company, USA], inverted microscope (Olympus, Japan), Infinite 200 PRO microplate reader (Tecan, Switzerland), flow cytometry (Beckman Coulter, USA), and thermal cycle PCR, real-time fluorescence quantitative PCR (qRT-PCR), gel imager, and electrophoresis apparatus (Bio-Rad Company, USA).

### Cell culture

The human gastric cancer cell line SGC7901 was purchased from Hubei Baios Biotechnology Co. Ltd. Tianmen, China. Cells were cultured in an incubator (37°C, 5% CO_2_). Dulbecco’s modified Eagle medium supplemented with 10% inactivated fetal bovine serum and antibiotics (100 U/ml of penicillin and 10 mg/L of streptomycin) was used for the cell culture. This study was approved by the Research Ethics Committee of Wuhan Polytechnic University (No. WHPU20211105).

### Prediction and screening of β-PGG and GC targets

The 2D structure of β-PGG was retrieved from the PubChem database (https://pubchem.ncbi.nlm.nih.gov/). The SDF file of the 2D structure was uploaded to the PharmMapper tool to predict the drug targets. The duplicated GeneCards (http://www.genecards.org/) were merged to obtain GC-related targets.

### Identification of intersection genes and construction of the PPI network

The target map for GC and β-PGG was generated using JVENN (https://bioinfogp.cnb.csic.es/tools/venny/). Next, the output was imported into the STRING database (https://string-db.org/) to construct the protein–protein interaction network. “*Homo sapiens*” was chosen as the target species and the minimum required interaction score for protein interactions was 0.4. Cytoscape was used to visualize and analyze the network. The top 8 target genes were selected according to the degree.

### Biological process and pathway enrichment analysis

The DAVID database (https://david.ncifcrf.gov/) was used to conduct Gene Ontology (GO) and Kyoto Encyclopedia of Genes and Genomes (KEGG) pathway enrichment analyses based on the common genes between GC-related genes and β-PGG-related genes. The threshold was set at *p<*0.05.

### Molecular docking analysis

Molecular docking was performed to explore the interactions between the drugs and the target proteins. The SDF file of the β-PGG structure was uploaded to Chem3D 17.0 software to generate the 3D structure. The 3D structure of the screened eight target genes was retrieved from the PDB database (http://www.rcsb.org/). AutoDock Vina was used to dock β-PGG on GC molecules to explore the binding pose and interactions between the drug and the target protein.

### CCK-8 assay

SGC7901 cells in the logarithmic growth stage were plated in 96-well plates at a density of 4 × 10^3^ cells/well and cultured for 24 h. Cells in the different groups were treated with β-PGG at 12.5, 25, 50, 100, and 200 μg/ml for 24 and 48 h. The CCK-8 assay was conducted to determine the inhibitory rate of the compound on SGC7901 cells. 5-Fluorouracil (5-FU) was used as the positive control. Each group set comprised five parallel wells. The culture medium was added into the control hole and incubated for 24 and 48 h. The culture medium in the hole was changed and 10 μl of CCK-8 was added per well then incubated under CO_2_ (5%, 37°C) for 3 h. The absorbance of the cell medium was read at 450 nm using an enzyme labeling instrument. The concentration with 100 μg/ml inhibition rate was selected for the subsequent studies.


$$\;Cell\;inhibition\;rate\;\left(\%\right)\;=\frac{OD_{\beta -PGG}-OD_{blank}}{OD_{negative\;control}-OD_{blank}}\times 100$$

### Cell cycle assay

Cells in the logarithmic growth stage were plated in six-well plates, a 2-ml medium was added to each well, and cells were cultured for 24 h and treated with different concentrations (0, 12.5, 25, 50, 100, and 200 μg/ml) of β-PGG for 48 h. The cells were digested with trypsin and centrifuged at 1,500 rpm and 4°C for 5 min. PBS (4°C) was used to wash the cells twice, after which they were fixed using 75% ethanol (4°C) at 4°C overnight. The fixed cells were collected by centrifugation and suspended in 400 μl of binding buffer at a concentration of about 10^6^ cells/ml. Propidium iodide (PI) staining solution (100 μl) was then added to the cells. Cells and PI were gently mixed and placed at 4°C under dark conditions for 30 min. The cell cycle was detected by flow cytometry.

### Annexin V-FITC/PI cell apoptosis assay

Human gastric cancer cells (SGC7901 cell line) at the logarithmic growth stage were plated in a six-well plate. The cells were cultured for 24 h and treated with different concentrations of β-PGG for 48 h. After incubation, the cells were digested using trypsin without EDTA, centrifuged, and collected. They were washed twice with 4°C PBS and then suspended in 400 μl of Annexin V at a density of 10^6^ cells/ml. Subsequently, 5 μl of Annexin V-FITC was added and mixed gently. The cells were incubated at 4°C in the darkness for 15 min. After incubation, 10 μl of PI was added, mixed gently, and incubated in the dark (4°C, 5 min). After 1 h, the apoptosis rate was calculated by flow cytometry.

### Mitochondrial membrane potential detection

Cells were pretreated as described in Section 2.3. Different concentrations of β-PGG were added to the cells and incubated for 48 h. In addition, 1 ml of JC-1 staining solution was added and mixed well. Then the mixture was incubated in a cell incubator at 37°C for 20 min. The supernatant was obtained after incubation and washed twice with JC-1 staining buffer (1×). Furthermore, 2 ml of cell culture medium was added to the cells, and changes in mitochondrial membrane potential in cells were examined by flow cytometry.

### Analysis of intracellular Ca^2+^ concentration

Intracellular Ca^2+^ concentration was determined by Fluo-3/AM assay. Cells were pretreated as described in Section 2.3. β- PGG at different concentrations was added to the cells and incubated for 48 h. Subsequently, 100 μl of Fluo-3/AM solution was added to the cells. Cells were incubated for 20 min and then 1 ml of Hanks’ balanced salt solution (HBSS) was added. The mixture was incubated in the dark for 40 min. Fluorescence intensity was recorded at an excitation/emission wavelength of 488/526 nm.

### qRT-PCR

SGC7901 cells were incubated with β-PGG (100 μg/ml) for 24 h. The mRNA levels of apoptosis-related genes (*P53*, *P21*, *CASP3*, *CASP9*, *Cytochrome C*, *BAX*, *BCL-2*, *IGF-BP3*, *PERP*, and *PUMA*) were determined by qPCR. Total RNA was extracted using the TRIzol reagent according to the manufacturer’s instructions. The RNA was reverse-transcribed using reverse transcription kit to obtain cDNA. qRT-PCR was performed with a two-step reaction process. The typical cycling condition was 95°C for 30 s followed by 40 cycles at 95°C for 5 s and 60°C for 30 s. β-Actin was used as the internal control. Relative gene expression levels were calculated by the 2^−ΔΔCT^ method. The primers used for qPCR are presented in **Table 1**.

**Table 1:** PCR-amplified primer sequences.

Gene	Forward primer (5′→3′)	Reverse primer (5′→3′)
*PUMA*	GAGGAGGAACAGTGGGCC	GGAGTCCCATGATGAGATTGT
*BAX*	AAGAAGCTGAGCGAGTGTCT	GTTCTGATCAGTTCCGGCAC
*BCL-2*	GCCTTCTTTGAGTTCGGTGG	GAAATCAAACAGAGGCCGCA
*CASP3*	ACTGGACTGTGGCATTGAGA	GCACAAAGCGACTGGATGAA
*CASP9*	GCCCCATATGATCGAGGACA	CAGAAACGAAGCCAGCATGT
*P21*	GACACCACTGGAGGGTGACT	CAGGTCCACATGGTCTTCCT
*P53*	GTTCCGAGAGCTGAATGAGG	TCTGAGTCAGGCCCTTCTGT
*IGF-BP3*	CCTGCC GTAGAGAAATGGAA	AGGCTGCCCATACTTATCCA
*PERP*	TGCCATCATTCTCATTGCAT	AACCCCAGTTGAACTCATGG
*Cytochrome C*	ATGAAGTGTTCCCAGTGCCA	CTCTCCCCAGATGATGCCTT
*β-Actin*	CATCCGCAAAGACCTGTACG	CCTGCTTGCTGATCCACATC

### Western blot analysis

Cells growing at the logarithmic growth stage were digested with trypsin to prepare a single-cell suspension. The concentration of the cell suspension was determined using a hemocytometer. The cells were then diluted to the corresponding volume with the culture medium and fully mixed to obtain a cell concentration of 1 × 10^5^ cells/ml. The cells were inoculated at a density of 2.5 × 10^5^ cells into a 6-cm plate. Furthermore, 2.5 ml of the cell suspension was inoculated into the 6-cm plate and incubated in a CO_2_ incubator (37°C, 5%) for 24 h. The β-PGG (100 μg/ml) solution was added into the culture dish for 24 h and then the culture medium was discarded. The cells were digested with trypsin, washed twice with 1 ml of PBS, and transferred to a 1.5-ml centrifuge tube. Subsequently, 100 μl of cell lysate (10 mm of Tris–HCl, pH 8.0, 1 mm of EDTA, 20% SDS, 5 mm of DTT, 10 mm of PMSF) was obtained by lysing the cells on ice for 30 min and sonicating 10 times for 2 s each time. The cell suspension was centrifuged at 12,000 rpm and 4°C for 5 min. The supernatant containing the protein was collected and the protein concentration was determined using a BCA protein quantitative kit. Furthermore, one-fourth of the sample volume of the protein loading buffer was added into the protein sample. The proteins were denatured at 95°C for 5 min and centrifuged at 12,000 rpm and 4°C for 5 min after cooling at room temperature. The protein samples were transferred to 10% SDS-polyacrylamide gel for electrophoresis separation. The conditions for the film were set at 100 V and 90 min under an ice bath. The PVDF membrane was sealed with TBST (5% skimmed milk) at room temperature for 1 h and incubated with monoclonal antibody overnight at 4°C. The membrane was washed three times with TBST for 5 min in every wash. The membrane was incubated with secondary antibodies at room temperature for 1 h. The membrane was washed three times with TBST for 5 min at each wash. ECL was added and the luminescent substrate was scanned using a gel imager.

### Statistical analysis

All data were expressed as mean ± standard deviation (SD). Statistical analysis and graphs were generated using GraphPad Prism 8.0.2 software. Any *p*-value less than 0.05 was considered significant.

## Results

### Construction of the “GC–target–PGG” network

**Figure 1:**
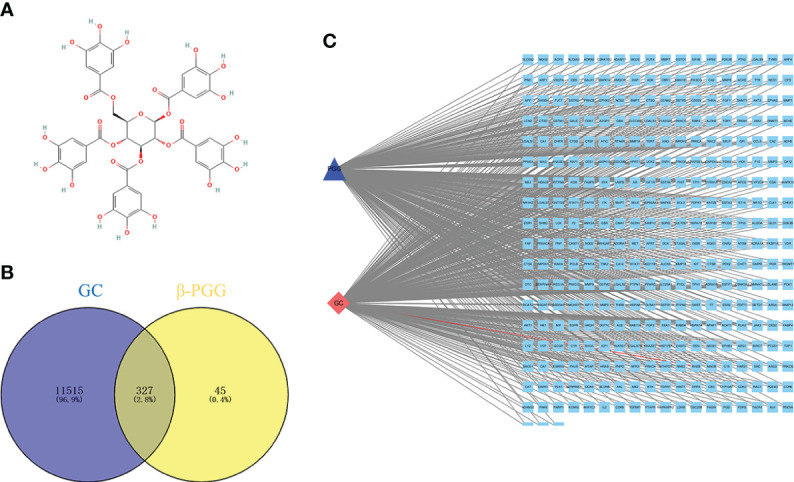
Pharmacological networks of 1,2,3,4,6-pentagalloyl-β-D-glucose (β-PGG) and gastric cancer. **(A)** The 2D structure of β-PGG. **(B)** Venn diagram showing the drug–disease interactions. **(C)** The “GC–target–PGG” network.

The 2D structure of β-PGG was retrieved from the PubChem database (**Figure 1A**). A total of 372 genes related to β-PGG were identified using the PharmMapper database. Furthermore, 11,842 GC-related genes were retrieved from the GeneCards database. A total of 327 common genes were obtained from the intersection of the drug-related genes and disease-related genes (**Figure 1B**). This implies that β-PGG modulates the progression of GC through these common genes. A “GC–target–PGG” network was constructed using the Cytoscape software to further explore the role of PGG in GC (**Figure 1C**).

### Construction of the PPI network and identification of hub genes

**Figure 2:**
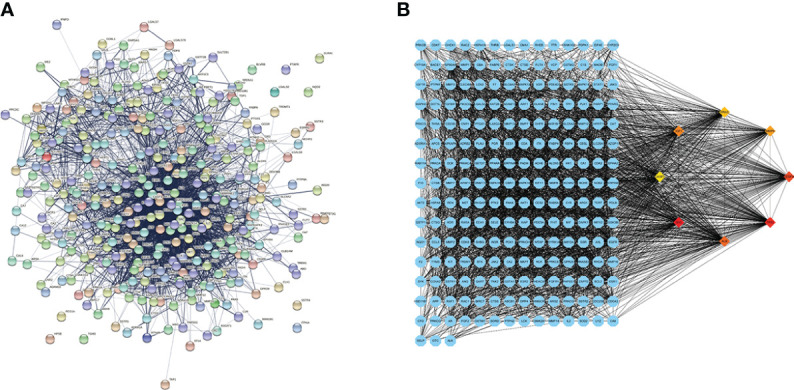
Protein–protein interaction (PPI) network diagram and the identified hub genes. **(A)** PPI network. **(B)** PPI map showing key genes; changes are presented based on the size and color of degree value.

The common genes were exported to the STRING database for the analysis of protein–protein interactions. A protein interaction network with 327 nodes and 3,430 edges was obtained. The protein interaction map was uploaded to Cytoscape for visualization (**Figure 2A**). Analysis of the network using the MCC calculation method in cytoHubba plug-in showed that the top 8 key target genes in the network were *CASP3*, *SRC*, *TP53*, *IGF1*, *VEGFA*, *MMP9*, *HRAS*, and *ALB* (**Figure 2B**).

### GO and KEGG pathway analyses

**Figure 3:**
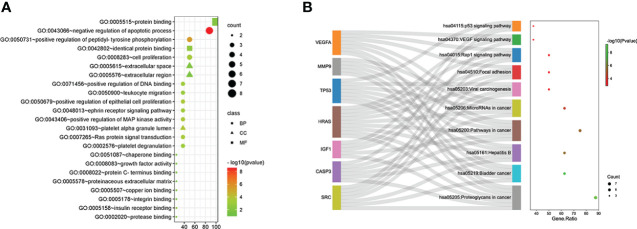
Gene Ontology (GO) and Kyoto Encyclopedia of Genes and Genomes (KEGG) pathway analyses of hub genes. **(A)** GO analysis. **(B)** KEGG pathway analysis.

GO and KEGG analyses of the eight hub genes were performed using R software to further explore the mechanism of β-PGG on GC. GO analysis showed that the genes were implicated in negatively regulating apoptosis and positively regulating DNA binding (**Figure 3A**). KEGG pathway analysis showed that these genes were associated with the P53, VEGF, and other signaling pathways (**Figure 3B**).

### Molecular docking results

**Figure 4:**
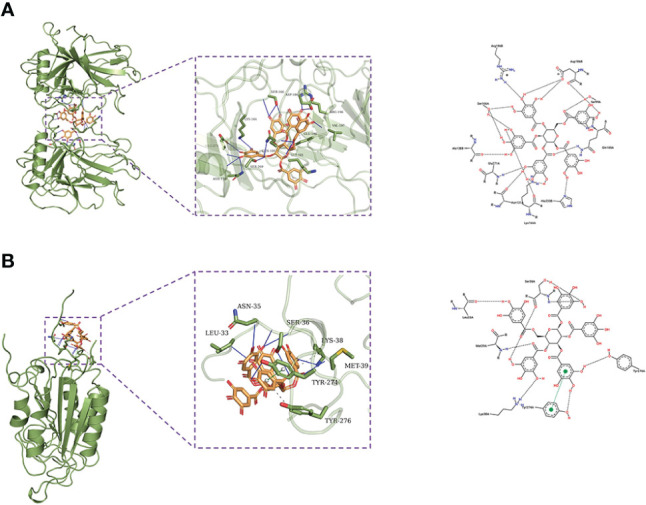
Docking poses of the drug on different targets. **(A)** *TP53*–β-PGG complex. **(B)** *CASP3*–β-PGG complex.

β-PGG was docked against the eight targets identified in Section 3.2. The binding energies for the complexes between the eight targets and β-PGG were less than −7 kcal/mol (**Table 2**). This indicates that β-PGG had a high binding affinity with the selected target. The protein–protein interactions showed that β-PGG inhibits GC by modulating the p53 signaling pathway. *TP53* and *CASP3* are the key genes that regulate apoptosis in the p53 pathway. The binding energy value of *TP53* and β-PGG was −10.1 kcal/mol (**Figure 4A**), whereas that of *CASP3* and β-PGG was −8.2 kcal/mol (**Figure 4B**), which were both less than −8 kcal/mol. This indicates high binding affinity. The p53 signaling pathway was selected for subsequent experimental verification.

**Table 2:** Molecular docking of the core target genes with PGG.

Ligand	Gene	Protein	Vina score	Cavity size	Center			Size		
					*x*	*y*	*z*	*x*	*y*	*z*
β-PGG	*SRC*	2SRC	−9.9	405	11	19	59	24	24	24
	*HRAS*	6Q21	−9.2	622	−16	73	48	24	24	24
	*IGF1*	2DSQ	−8.7	1,792	14	−15	−32	24	24	24
	*MMP9*	1L6J	−7.9	241	31	56	53	24	24	24
	*CASP3*	5i9b	−8.2	112	1	−5	−20	24	24	24
	*TP53*	1ma3	−10.1	1,951	13	5	71	24	24	24
	*VEGFA*	3v2a	−7.7	619	39	−10	12	35	24	24
	*ALB*	1bj5	−9.6	8,563	45	11	16	35	32	30

### β-PGG inhibits the proliferation of SGC7901 cells

**Figure 5:**
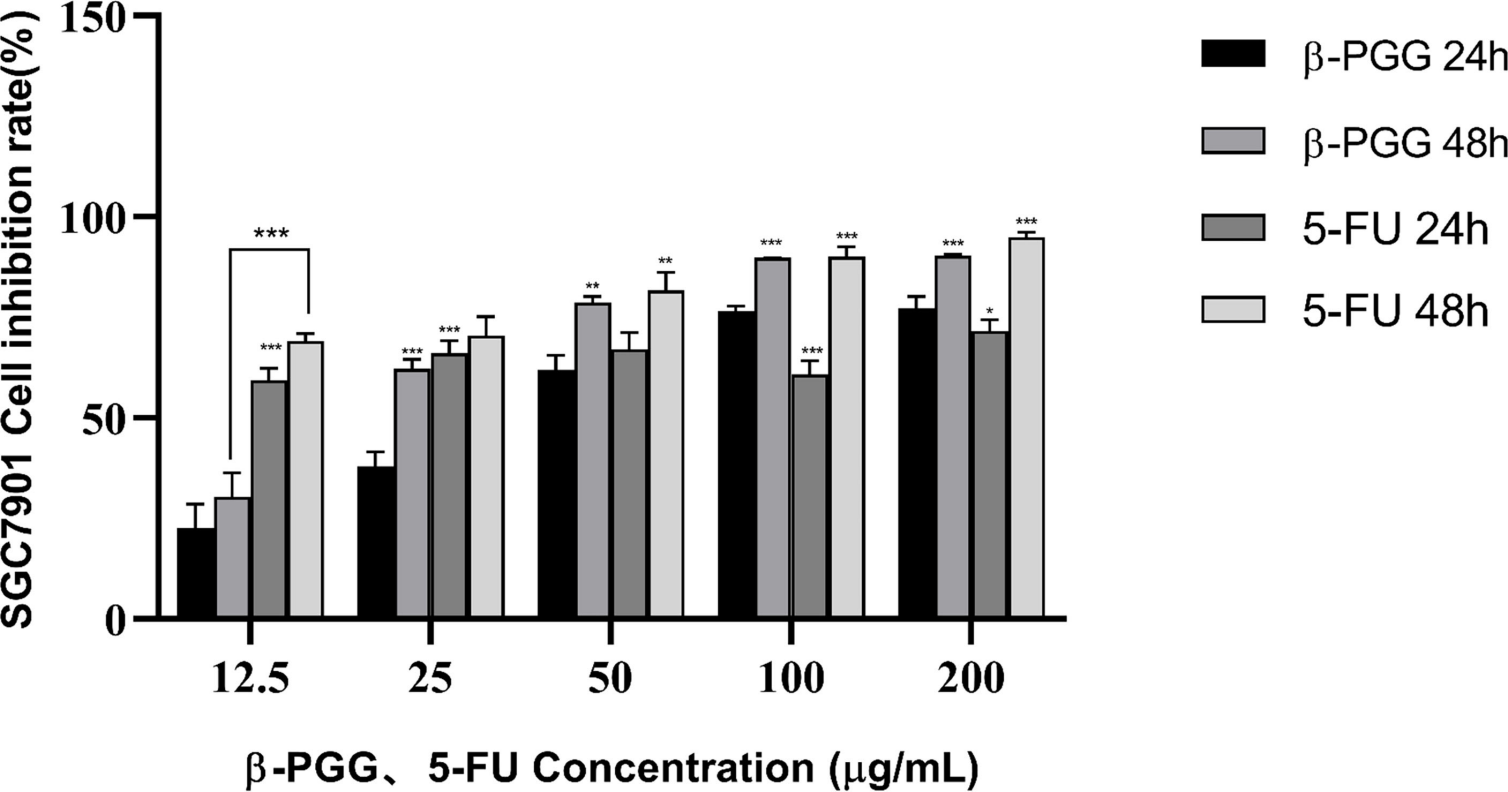
Effect of β-PGG on the proliferation of SGC7901 cells. SGC7901 cells were treated with different concentrations of β-PGG and different concentrations of 5-FU (12.5, 25, 50, 100, and 200 μg/ml) for 24 and 48 h and detected by the CCK-8 assay. *p < 0.05, **p < 0.01, ***p < 0.001 vs. β-PGG 24 h.

The results showed that β-PGG inhibited the proliferation of SGC7901 cells. The inhibition rate of SGC7901 increased with the increase in β-PGG concentration. When the concentrations were 100 and 200 μg/ml, the inhibition rate of β-PGG was significantly stronger compared with that of 5-FU for 24 h (**Figure 5**). The findings showed that the inhibition rate of β-PGG at a concentration of 50 μg/ml was the inhibitory rate of 5-FU against SGC7901. The IC_50_ values of β-PGG and 5-FU were 38.36 and 19.30 μg/ml, respectively (**Table 3**).

**Table 3:** IC_50_ values of compounds on SGC7901 cells.

Compound	Time (h)	IC_50_ value (μg/ml)
β-PGG	24	38.86
	48	19.3
5-FU	24	9.12
	48	4.06

### β-PGG modulates the gastric cancer cell cycle

**Figure 6:**
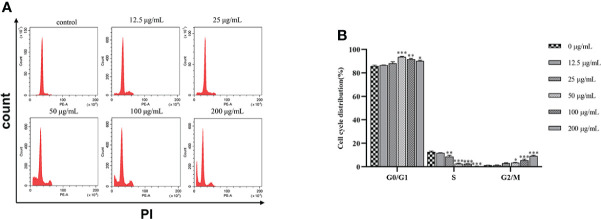
Effects of 1,2,3,4,6-pentagalloyl-β-D-glucose on the SGC7901 cell cycle. **(A)** Intracellular fluorescence intensity of SGC7901 cells cultured with 1,2,3,4,6-pentagalloyl-β-D-glucose (0, 12.5, 25, 50, 100, and 200 µg/ml) for 48 (h) **(B)** Average fluorescence intensity of SGC7901 cells. *p < 0.05, **p < 0.01, ***p < 0.001 vs. 0 µg/ml.

SGC7901 cells were treated with β-PGG for 48 h (**Figure 6**). The proportion of cells in the G0/G1 and G2/M phases increased when the dose of β-PGG was 12.5 μg/ml, whereas the number of cells in the S phase decreased compared with the number in the control group. Treatment of SGC7901 cells with β-PGG at a concentration of 50 μg/ml significantly increased the proportion of cells in the G0/G1 phase and decreased the number of cells in the S phase, whereas the proportion of cells in the G2/M phase did not change. These findings indicate that the low concentration of β-PGG inhibited the G0/G1 phase of GC cells. The high concentration of β-PGG inhibited the G2/M phase of GC cells.

### Effect of β-PGG on apoptosis of tumor cells

**Figure 7:**
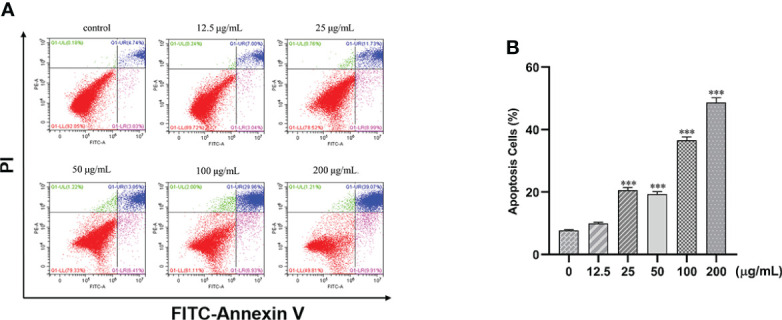
Effects of 1,2,3,4,6-pentagalloyl-β-D-glucose on the apoptosis of SGC7901 cells. **(A)** FITC-Annexin V/PI double-staining flow cytometry showing the apoptosis rate of SGC7901 cells after treatment with 1,2,3,4,6-pentagalloyl-β-D-glucose and 5-FU (0, 12.5, 25, 50, 100, and 200 µg/ml) for 48 h **(B)** Average apoptosis rate of SGC7901 cells. ***p < 0.001 vs. 0 µg/ml.

Treatment of cells with different concentrations of β-PGG (12.5, 25, 50, 100, 200 μg/ml) for 48 h increased the proportion of apoptotic cells in a concentration-dependent manner (**Figure 7**). Treatment of SGC7901 cells with 25, 50, 100, and 200 μg/ml of β-PGG significantly increased the apoptosis rate (*p*< 0.001). The early apoptosis rate of SGC7901 cells at 200 μg/ml of β-PGG was 9.91%, and the rate of late apoptosis was 39.07%.

### β-PGG decreases the mitochondrial membrane potential of tumor cells

**Figure 8:**
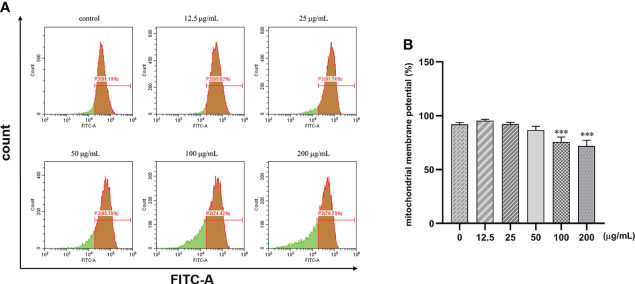
Effects of 1,2,3,4,6-pentagalloyl-β-D-glucose on mitochondrial membrane potential in SGC7901 cells. SGC7901 cells were incubated with different concentrations of 1,2,3,4,6-pentagalloyl-β-D-glucose for 48 (h) Δψm was evaluated using JC-1 in treated cells. **(A)** Δψm after treatment of cells with 0, 12.5, 50, 100, and 200 μg/ml of β-PGG. **(B)** Average mitochondrial membrane potential rate of SGC7901 cells. ***p < 0.001 vs. 0 µg/ml.

Human gastric cancer SGC7901 cells were treated with different concentrations of β-PGG for 48 h. We found that β-PGG treatment decreased the mitochondrial membrane potential of SGC7901 cells (**Figure 8**). Treatment of cells with β-PGG concentrations above 100 μg/ml increased the proportion of cells with decreased mitochondrial membrane potential compared with the proportion of cells in the control group (*p*< 0.001) in a concentration-dependent manner. Notably, 200 μg/ml of β-PGG reduced the mitochondrial membrane potential of SGC7901 cells by 20.4%. These findings show that β-PGG decreases mitochondrial membrane potential and increases mitochondrial membrane permeability of SGC7901 cells.

### β-PGG increases Ca^2+^ concentration in tumor cells

**Figure 9:**
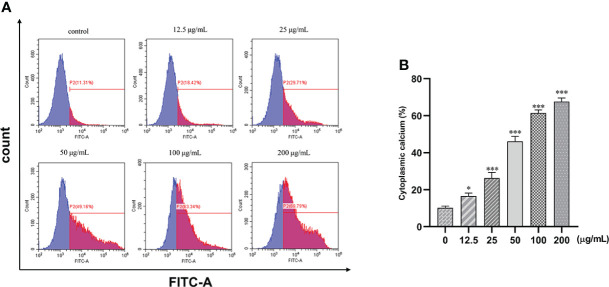
Effect of 1,2,3,4,6-pentagalloyl-β-D-glucose on intracellular Ca^2+^ concentration in SGC7901 cells. SGC7901 cells were incubated with different concentrations of 1,2,3,4,6-pentagalloyl-β-D-glucose for 48 (h) Fluo-3/AM assay was performed to determine Ca^2+^ concentration. **(A)** Intracellular Ca^2+^ concentration after treatment of cells with 0, 12.5, 50, 100, and 200 μg/ml of β-PGG. **(B)** Average cytoplasmic calcium rate of SGC7901 cells. *p < 0.05, ***p < 0.001 vs. 0 µg/ml.

The fluorescence intensity of the cells increased with the increase in concentration. Intracellular calcium concentration significantly increased by 51% after treatment with 25, 50, 100, and 200 μg/ml of β-PGG compared with the control group (*p*< 0.001) (**Figure 9**). This implies that β-PGG-mediated apoptosis of SGC7901 cells is closely related to the increase of intracellular free Ca^2+^ concentration.

### β-PGG modulates the expression of apoptosis-related genes in tumor cells

**Figure 10:**
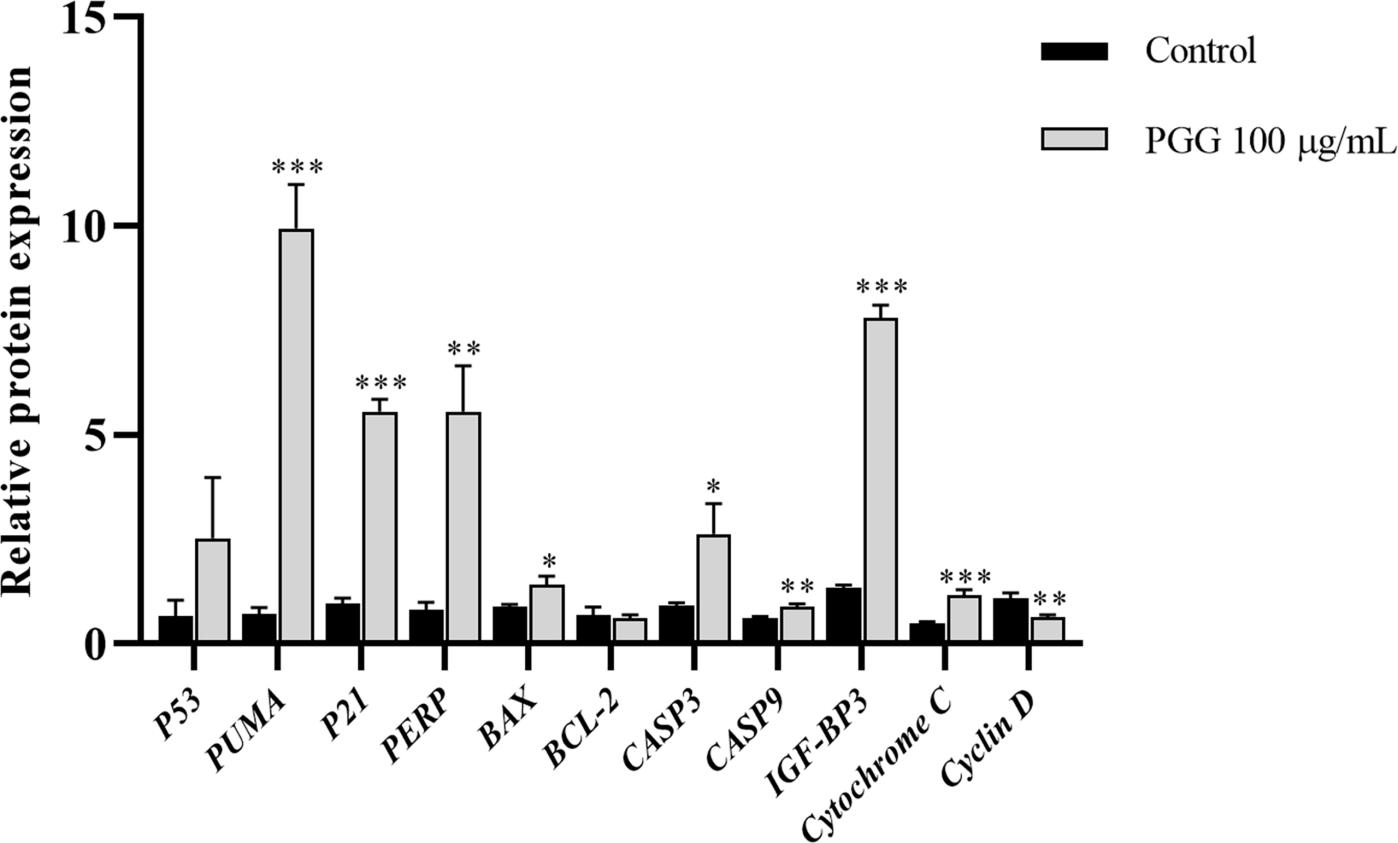
The expression levels of apoptosis-related genes in SGC7901 cells. qRT-PCR was used to determine the mRNA levels of apoptosis-related genes in SGC7901 cells. The expression levels of *P21*, *PERP*, *IGF-BP3*, *PUMA*, *BAX*, *BCL-2*, *CASP9*, *Cyclin D*, *Cytochrome C*, *CASP3*, and *P53*. Cells were treated with 100 µg/ml of 1,2,3,4,6-pentagalloyl-β-D-glucose for 24 h. *p < 0.05, **p < 0.01, ***p < 0.001 vs. control.

The mRNA expression levels of *P21*, *PUMA*, *IGF-BP3*, *CASP3*, and *Cytochrome C* were significantly higher in the treatment groups compared with the expression levels of the control group (*p*< 0.001); the expression levels of *PERP* and *CASP9* in the treatment groups increased significantly compared with the expression levels of the control group (*p*< 0.01); and the mRNA expression levels of *BAX* and *CASP3* also increased significantly (*p*< 0.05). Notably, the mRNA expression level of *Cyclin D* was lower in the treatment groups compared with the expression level in the control group (*p*< 0.01) (**Figure 10**). These findings show that β-PGG modulated the mRNA expression levels of apoptosis-related genes of SGC7901 cells.

### Effect of β-PGG on the protein expression in tumor cells

**Figure 11:**
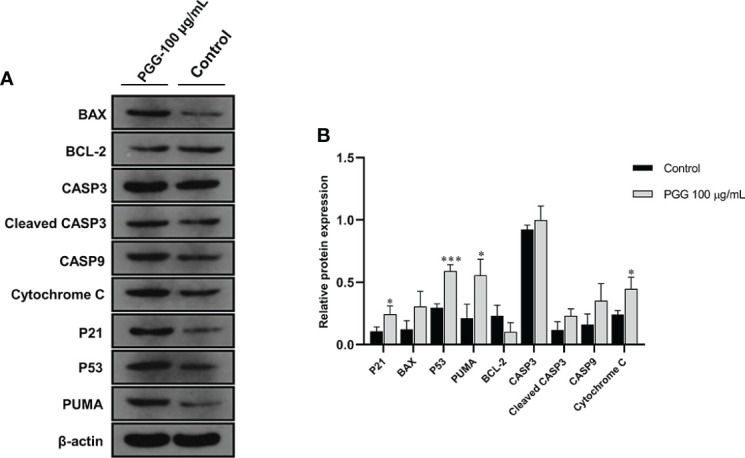
The expression levels of apoptosis-related proteins in SGC7901 cells. **(A)** The expression levels of BAX, BCL-2, CASP3, Cleaved CASP3, CASP9, Cytochrome C, P21, P53, and PUMA in SGC7901 cells as determined by Western blot. Cells were treated with 100 µg/ml of 1,2,3,4,6-pentagalloyl-β-D-glucose for 24 h **(B)** The average band grayscale of P21, BAX, P53, PUMA, BCL-2, PUMA, CASP3, Cleaved CASP3, CASP9, and Cytochrome C proteins. Cells were treated with 100 µg/ml of 1,2,3,4,6-pentagalloyl-β-D-glucose for 24 h *p < 0.05, ***p < 0.001 vs. control.

Treatment of SGC7901 cells with β-PGG for 24 h increased the protein level of P53 compared with the control group (**Figure 11**). Western blot analysis showed that the protein content of P21 implicated in the cell cycle arrest was increased by the treatment of SGC7901 cells with β-PGG compared with the control (*p*< 0.05). The intracellular expression of P53 protein was higher in β-PGG-treated cells compared with the control group (*p*< 0.001). Moreover, β-PGG treatment increased the expression of Cytochrome C and PUMA (*p*< 0.05). The release of Cytochrome C in the mitochondria was regulated by several proteins. The results showed that the expression of pro-apoptotic protein BAX was upregulated, whereas that of anti-apoptotic protein BCL-2 was downregulated. Furthermore, the ratio of BAX to BCL-2 was increased.

## Discussion

β-PGG is a compound extracted from the water chestnut shell. Studies have demonstrated that β-PGG blocks the cell cycle, induces apoptosis, and inhibits tumor development. A previous study showed that β-PGG had stronger cytotoxic effects against cancer cells compared with gallic acid (30). β-PGG was reported to decrease the expression of GNMT in hepatocellular carcinoma by downregulating the expression of Myc (31). *In-vitro* studies showed that β-PGG exerts anticancer activities by promoting apoptosis, antiproliferation, antiangiogenesis, antimetastasis, and inhibition of glycoproteins (30, 32). Kant et al. (31) reported that PGG inhibits the expression of Myc at the mRNA and protein levels in HepG2 cells. Li etal. (33) conducted several *in-vivo* experiments, and the findings showed that β-PGG has inhibitory activity against prostate cancer, lung cancer, and breast cancer cells. Kawk etal. (17) reported that β-PGG upregulates the expression of the tumor suppressor gene *P53* and the expression of *P21*, thus inhibiting colon cancer. However, the mechanism of β-PGG against GC has not been fully elucidated. The aim of this study was to explore the targets of β-PGG for the treatment of GC. In addition, the effect of β-PGG on the proliferation and apoptosis of SGC7901 cells was explored. Moreover, the mechanism of network pharmacological prediction and its relationship with the p53 signaling pathway were elucidated.

Network pharmacology is a new technique for multitarget drug molecular design based on the systems biology theory, biological system network analysis, and specific signal node selection (25). This technique is used to explore the relationship between drugs, targets, and diseases, to predict the mechanism of action of drugs against various diseases. GC patients have a poor prognosis. Therefore, a pharmacological network approach was used to explore whether β-PGG inhibits the proliferation of GC cells. A “GC–target–PGG” network was constructed to explore the various effects of β-PGG on GC. The GO and KEGG pathway analyses showed that β-PGG modulated GC cell cycle progression and apoptosis of GC cells. These activities were mediated through multiple cancer-related signaling pathways. The top 8 genes with the highest correlation with GC (*CASP3*, *SRC*, *TP53*, *IGF1*, *VEGFA*, *MMP9*, *HRAS*, and *ALB*) were selected. Binding of β-PGG to the eight hub genes was predicted through molecular docking. β-PGG showed high binding affinity against *TP53* and *CASP3*; therefore, they were selected for subsequent analyses. Analysis of the binding poses of β-PGG showed that it formed hydrogen bond interactions with *TP53* and *CASP3*. *In-vitro* experiments were conducted to validate the bioinformatics results on the effect of β-PGG on GC cells.

The effect of β-PGG on mitochondrial membrane potential and intracellular Ca^2+^ concentration of cells was determined to further explore the mechanism of β-PGG in inhibiting apoptosis. Mitochondrial membrane potential decreased, whereas intracellular Ca^2+^ concentration increased with the increase in drug concentration. In addition, changes in the expression of p53 signal pathway-related genes and proteins were explored by qRT-PCR and Western blot analyses. The results showed that β-PGG treatment significantly upregulated the mRNA expression of *P21*, *PUMA*, *PERP*, *IGF-BP3*, *CASP3*, *CASP9*, *Cytochrome C*, *BAX*, and *P53* genes in GC cells. Western blot results showed that β-PGG increased the expression levels of *Cytochrome C*, *CASP9*, *P21*, *PUMA*, *BAX*, and *Cleaved CASP3*, which are directly regulated by P53. Notably, *BCL-2* expression was downregulated. Studies have shown that the p53 signaling pathway plays an important role in apoptosis (30). Activation of this pathway promotes cell angiogenesis and inhibits apoptosis and DNA repair, leading to the occurrence and progression of cancer. The cell cycle regulatory factors include cyclin, cyclin-dependent kinase (CDK), and cyclin-dependent kinase inhibitor (CDKI), which constitute a regulatory network. With CDK as the core, cyclin promotes cell proliferation, whereas CDKI suppresses cell proliferation and synchronal segmentalization of cell cycle progression. As a broad-spectrum CDKI, P21 preferentially binds to CDK2 and CDK4 and inhibits their activity, whereas p21 inhibits the activity of PCNA. There is also a direct inhibition of DNA synthesis (34). *CyclinD1* is the promoter of cell cycle progression and the *CyclinD1* gene is a proto-oncogene, which interacts with a variety of oncogenes to promote cell transformation. P21 inhibits the activity of CyclinD1, leading to the arrest of the cell cycle and inhibition of cell proliferation and cell growth. The results showed that the *P21* gene and protein were upregulated, whereas the *CyclinD* gene was downregulated, indicating that PGG inhibited the cell cycle to prevent the occurrence and development of gastric cancer.

In summary, the present findings show that β-PGG exerts antitumor effects *in vitro* by modulating the cell cycle. Specifically, β-PGG arrests the SGC7901 cell cycle at the G1 phase by upregulating the expression of P21. In addition, β-PGG exerts antitumor effects by inducing tumor cell apoptosis. The expression level of *P53* in SGC7901 cells increased after treatment with β-PGG. Upregulation of P53 in cells promotes the expression of PUMA, BAX, and other apoptosis-related proteins. Upregulation of PUMA and elevation of the BAX to BCL-2 ratio increase the permeability of the mitochondrial outer membrane leading to the release of Cytochrome C. This ultimately activates CASP3 and CASP9, thereby promoting apoptosis.

In the future, the effects and toxicity of β-PGG *in vivo* need to be further verified. This study provides new ideas and a theoretical basis for the subsequent research and development of antigastric cancer drugs.

## Data availability statement

The datasets presented in this study can be found in online repositories. The names of the repository/repositories and accession number(s) can be found in the article/**Supplementary material**.

## Author contributions

Y-hJ drafted the manuscript. J-hB conducted the experiments. M-rW drew the figures. S-jY created and plotted the tables. L-mW, YY, and H-xW revised the manuscript. All authors contributed to the article and approved the submitted version.

## Funding

This work was supported by the Primary Research & Development Plan of Hubei Province (2020BBB074, 2020BBA046).

## Acknowledgments

We thank the home-for-researchers for editing the language of the manuscript.

## Conflict of interest

The authors declare that the research was conducted in the absence of any commercial or financial relationships that could be construed as a potential conflict of interest.

## Publisher’s note

All claims expressed in this article are solely those of the authors and do not necessarily represent those of their affiliated organizations, or those of the publisher, the editors and the reviewers. Any product that may be evaluated in this article, or claim that may be made by its manufacturer, is not guaranteed or endorsed by the publisher.

## Abbreviations

β-PGG, 1,2,3,4,6-Pentagalloyl-β-D-glucose; GC, gastric cancer; GO, Gene Ontology; KEGG, Kyoto Encyclopedia of Genes and Genomes; qRT-PCR, real-time fluorescence quantification polymerase chain reaction; DMEM, Dulbecco’s modified Eagle medium; PI, propidium iodide; EDTA, ethylenediaminetetraacetic acid; OD, optical density; SDS, sodium dodecyl sulfate; PBS, phosphate-buffered saline; 5-FU, 5-fluorouracil; IC_50_, median inhibition concentration; PVDF, polyvinylidene fluoride; DTT, dithiothreitol; PMSF, phenylmethanesulfonyl fluoride.
