# Supplementary material for: Investigation of the inhibition effect of 1,2,3,4,6-pentagalloyl-β-D-glucose on gastric cancer cells based on a network pharmacology approach and experimental validation
Source: Front Oncol. 2022 Aug 3;12:934958. doi: 10.3389/fonc.2022.934958 (PMC9383036; doi:10.3389/fonc.2022.934958)

1. Effects of different concentrations of β-PGG on the inhibition rate of hepatocellular carcinoma HepG2 cells

Compound	Time	HepG2 Cell growth inhibition rate (%)				
		12.5 μg/mL	25 μg/mL	50 μg/mL	100 μg/mL	200 μg/mL
β-PGG	24 h	6.26±2.7	42.33±1.95	68.49±0.31	77.23±0.97	83.8±0.89
	48 h	21.35±5	48.91±4.63	73.52±4	89.23±1.41	85.95±1.52
5-FU	24 h	39.38±6.05	45.52±3.58	50.03±2.4	57.1±3.27	60.18±0.93
	48 h	73.53±0.43	79.44±0.85	85.38±0.47	85.46±0.85	87.69±0.51

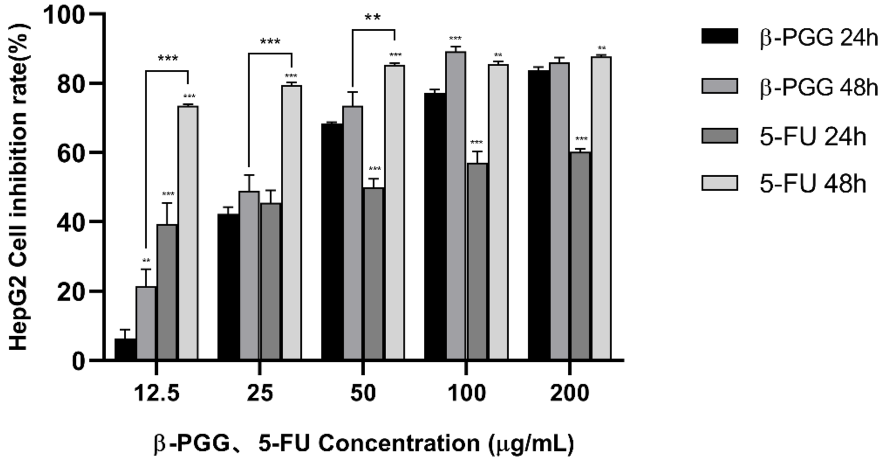

2. Effects of different concentrations of β-PGG on the inhibition rate of Mouse gastric cancer cell line MFC cells

Compound	Time	MFC Cell growth inhibition rate (%)					
		0 μg/mL	12.5 μg/mL	25 μg/mL	50 μg/mL	100 μg/mL	200 μg/mL
β-PGG	24 h	100.98±0.086	91.14±0.064	78.37±0.059	68.44±0.044	61.65±0.096	49.36±0.033
	48 h	100±0.084	67.51±0.072	51.87±0.020	46.84±0.052	29.22±0.050	22.79±0.044
5-FU	24 h	100.98±0.086	83.48±0.097	77.5±0.084	66.22±0.101	55.76±0.080	53.34±0.065
	48 h	100±0.084	57.76±0.058	45.7±0.035	44.17±0.052	33.33±0.020	22.08±0.055

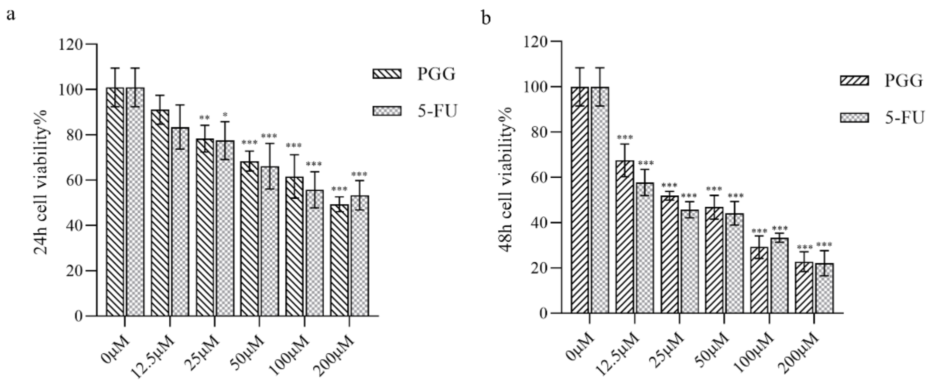

Supplement: Supplementary file 1 [file DataSheet_1.pdf]
